# Immunohistochemical Markers of Mitochondrial Electron Transport Chain Instability in Human Brain Regions: A Study of Aging and Alzheimer’s Disease

**DOI:** 10.3390/ijms27062816

**Published:** 2026-03-20

**Authors:** Tatiana I. Baranich, Vladimir S. Sukhorukov, Olga V. Velts, Dmitry N. Voronkov, Ekaterina V. Shcherbak, Anna V. Egorova, Alexander S. Romanenko, Dmitry S. Lazarev, Alexander P. Raksha, Irina G. Charyeva, Alexander N. Yatskovskiy, Valeria V. Glinkina, Sergey N. Illarioshkin

**Affiliations:** 1 Laboratory of Neuromorphology, Russian Center of Neurology and Neurosciences, 125367 Moscow, Russia; 2 Department of Anatomy and Histology, I.M. Sechenov First Moscow State Medical University (Sechenov University), 125009 Moscow, Russia; 3 Department of Morphology, Pirogov Russian National Research Medical University, 117513 Moscow, Russia; 4 Pirogov City Clinical Hospital No. 1, 119049 Moscow, Russia

**Keywords:** Alzheimer’s disease, aging, mitochondria, oxidative stress, neuron, hippocampus

## Abstract

Expanding research indicates that oxidative stress, particularly mitochondrial oxidative stress, is one of the key components in the pathogenesis of Alzheimer’s disease (AD). Mitochondrial oxidative stress is largely driven by impaired function of electron transport chain (ETC) complexes and their regulators. This study conducted an immunohistochemical analysis of ETC proteins (α-subunit of complex V, subunits MTCO1 and MTCO2 of complex IV) and mitochondrial complex V inhibitor IF-1 in the neurons of the caudate nucleus head, hippocampus, anterior cingulate gyrus, middle frontal gyrus, and inferior parietal lobule using autopsy material from patients with sporadic AD. Comparisons were made with similar brain regions in autopsy material from age-matched elderly patients and young patients. The results revealed a pattern of ETC impairment in AD fundamentally distinct from that observed in physiological aging. Specifically, a hippocampus-specific failure of the adaptive response was identified: unlike other brain regions, compensatory upregulation of ATP synthase does not occur here despite critical reduction in the protective protein IF-1, directly explaining the heightened vulnerability of hippocampal neurons to damage. Our data deepen the understanding of AD pathogenesis by highlighting region-specific mitochondrial defects as promising targets for tailored therapeutic intervention.

## 1. Introduction

Alzheimer’s disease (AD) is the most common neurodegenerative disorder, currently representing a global challenge for worldwide healthcare. The incidence and prevalence of Alzheimer’s disease are increasing as the global population ages and overall life expectancy rises. According to WHO projections, by 2050 over 150 million people worldwide will have AD, exacerbated by the lack of therapies capable of curing or significantly slowing the disease progression.

The clinical manifestation of AD is progressive cognitive dysfunction, including memory loss. Morphological features of AD include both extracellular changes (formation of beta-amyloid plaques) and intraneuronal alterations, specifically the formation of neurofibrillary tangles composed of hyperphosphorylated tau protein. However, the pathogenesis of AD remains unclear despite numerous scientific hypotheses about the disease’s development, such as the amyloid cascade hypothesis, tau hypothesis, neuroinflammation theory, metal ion imbalance hypothesis, and oxidative stress theory [1,2,3].

Analysis of current concepts clearly demonstrates that despite the multitude of pathological pathways described in these theories, they all converge on a single key element—oxidative stress, which involves an imbalance between the formation and neutralization of reactive oxygen species (ROS) [4]. Although activation of pro-oxidant enzymes, disruption of transition metal homeostasis, and beta-amyloid itself contribute to this pathogenetic process, mitochondrial dysfunction remains the primary cause of oxidative stress in neurons. Most free radicals are generated as byproducts of the mitochondrial electron transport chain (ETC) [5]: electron transfer in complexes I, III, and IV is coupled with proton translocation across the inner mitochondrial membrane from the matrix to the intermembrane space, creating the membrane potential ΔΨm and pH gradient, which are utilized by complex V (ATP synthase) to produce ATP from ADP and phosphate. Mitochondrial dysfunction is often associated with increased reactive oxygen species (ROS) and increased lipid peroxidation, protein oxidation, and DNA damage, collectively triggering neurodegeneration. Despite antioxidant defense systems (glutathione system, SOD2) in neurons, these systems become dysfunctional and insufficient under chronic mitochondrial dysfunction, exacerbating damage [6,7].

In the early AD stages, impairments in complex IV of the electron transport chain have been described, associated with direct toxicity of beta-amyloid, oxidative modification of metal ions, and several other factors, leading to electron leakage and increased ROS production [8,9]. Some studies show selective reduction in complex IV activity in frontal, temporal, and parietal lobes in AD [10], while other publications demonstrate increased expression of complex IV compared to healthy individuals [11]. Importantly, complex IV of the electron transport chain is the terminal enzyme of direct electron transfer, and its activity serves as the primary regulator of electron flux through the entire chain. The stability and proper enzymatic function of this complex are largely determined by the quantitative ratio of complex IV subunit expression, particularly MTCO1 and MTCO2 encoded by mitochondrial DNA [12,13]. Correct structural organization of this complex enables the coupling of electron transfer with proton translocation and is essential for efficient ETC function. Conversely, improper subunit ratios may lead to impaired complex assembly and stability, accumulation of unutilized subunits and their degradation, and reduced ATP synthesis. It is known that AD shows decreased mRNA levels encoding these subunits in the middle temporal gyrus [10]. Another study revealed reduced RNA encoding the MTCO2 subunit of complex IV in the hippocampus in AD but found no changes in RNA of nuclear DNA-encoded complex IV subunits [12]. Paradoxically, research on MTCO1 subunit RNA levels showed their increase in 6- and 12-month-old transgenic AD mice exhibiting cognitive impairments from 3 months of age [14].

Dysfunction of complex IV of the ETC may reduce the proton gradient necessary for the operation of complex V, ATP synthase, which utilizes this gradient for ATP synthesis. It has been demonstrated that in AD, the lipoxidation of the α-subunit of complex V (attachment of 4-HNE groups to protein structures), leading to reduced activity of the entire mitochondrial complex V, was observed in the entorhinal cortex at Braak stages I/II, as well as in the hippocampus and parietal cortex of patients at Braak stages III–VI [15]. Reduced expression of some nuclear-encoded genes of ATP synthase was also detected in the posterior cingulate cortex, hippocampal CA1 field, middle temporal gyrus, and entorhinal cortex [16]. However, overall data on the role of ATP synthase in the AD pathogenesis remain extremely limited.

A decrease in the proton gradient may not only cause functional impairment of Complex V but also activate its reverse (ATPase) function, which involves ATP hydrolysis to restore the membrane potential ΔΨm; under prolonged hydrolytic activity, this can lead to ATP deficiency and further exacerbate oxidative stress [4,16]. Under ATP-deficient conditions, the hydrolytic activity of Complex V is suppressed by its inhibitor IF1: binding of IF1 to Complex V reduces the latter’s activity [17]. Notably, tissues with high metabolic rates (such as nervous tissue, cardiac muscle, and proximal renal tubular epithelium) exhibit elevated levels of IF1 protein and high IF1:ATP synthase expression ratios [18]. Interestingly, by binding to ATP synthase, IF1 stabilizes its dimerization, promoting the formation and maintenance of normal mitochondrial cristae architecture and density [18]. Current evidence indicates that the complex architecture of the mitochondrial inner membrane, particularly the formation of numerous cristae, depends on coordinated actions of specific proteins. ATP synthase dimers play a key role in maintaining the curvature of the inner mitochondrial membrane: their conformation stabilizes lipid bilayer bends, forming narrow isthmuses and tubular structures that serve as the foundation for future cristae. However, the formation of fully developed, functionally distinct cristae and alterations in their morphology require additional regulatory proteins. One such protein is OPA1, which is anchored to the mitochondrial inner membrane and performs multiple functions, including maintaining cristae structure and morphology and facilitating their pro-apoptotic remodeling. Thus, ATP synthase dimers create and sustain membrane curvature, while OPA1 organizes them morphologically into cristae, controls cristae integrity, and regulates their functional connection with the remaining membrane [19,20]. Additionally, the IF1 role in regulating mitochondrial morphology during apoptosis warrants special attention: increased IF1 expression has been shown to inhibit oxidative stress-induced proteolytic cleavage of OPA1, thereby restricting cristae remodeling and acting as an anti-apoptotic factor [21].

The neuroprotective effect of IF1 factor has been described in neurodegenerative diseases and various pathological conditions such as ischemia [22]. For instance, in vivo administration of IF1 factor in transgenic mice with Parkinson’s disease expressing human α-synuclein led to improved motor activity [23]. However, data on the role of IF1 protein in AD are extremely scarce. Several studies indicate an important role of the IF1 factor in regulating cognitive abilities, specifically memory [21]: genetic ablation of the IF1 factor in forebrain neurons impairs learning in mice, while its overexpression promotes long-term memory [24,25]. Thus, investigating the relationship between alterations in mitochondrial complexes IV and V and the IF1 factor in various brain regions of AD patients is highly relevant.

The study was aimed at immunohistochemically determine dysfunctional features of complexes IV and V of the mitochondrial electron transport chain in different brain regions of patients with Alzheimer’s disease using autopsy material.

## 2. Results

A comparative immunohistochemical analysis of markers for subunits of complex IV (MTCO1, MTCO2), the α-subunit of mitochondrial complex V, and its regulatory protein IF1 was performed in preserved neurons of layers 3 and 5 of the middle frontal and anterior cingulate gyri; head of the caudate nucleus; hippocampus (CA1 and CA2 fields); and layers 2, 3, 4, 5 of the inferior parietal lobule in patients with sporadic AD, elderly patients, and a young control group.

### 2.1. Middle Frontal Gyrus

**Figure 1:**
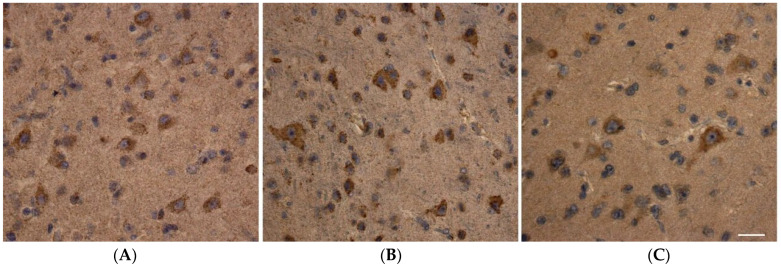
(**A**–**C**) Immunohistochemical distribution of the MTCO1 subunit marker in layer 5 neurons of the middle frontal gyrus. (**A**) Staining in young control group neurons; (**B**) staining in aging group neurons; (**C**) staining in AD patient neurons. Objective magnification: 40×. Scale bar = 25 μm.

During aging, layer 3 neurons of the middle frontal gyrus showed decreased levels of the mitochondrial complex V marker (*p* = 0.0207), while IF-1 marker levels remained unchanged compared to controls. MTCO1 marker levels increased (*p* = 0.021), whereas no significant changes in MTCO2 levels were detected. In layer 5, conversely, decreased levels of the IF-1 marker (*p* = 0.0206) were observed, while the levels of the complex V marker were unchanged. Changes in complex IV subunits were similar to those in layer 3: MTCO1 marker levels increased (*p* = 0.0082) (Figure 1), while no significant changes in MTCO2 levels were detected.

**Figure 2:**
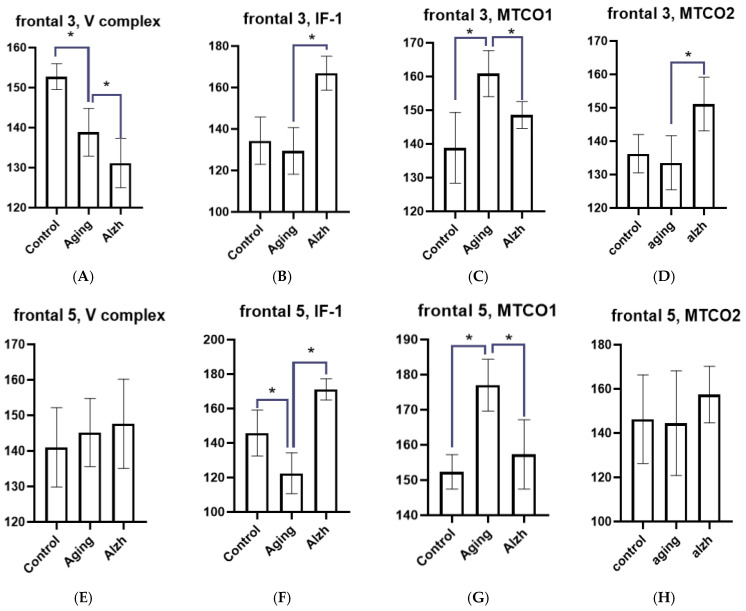
Results of morphometric analysis of immunohistochemical distribution for mitochondrial complex V markers, IF1 factor, and mitochondrial complex IV subunits (MTCO1 and MTCO2) in layer 3 and 5 neurons of the frontal gyrus. (**A**) Changes in the mitochondrial complex V marker in layer 3 neurons of the middle frontal gyrus. (**B**) Changes in the IF1 marker in layer 3 neurons of the middle frontal gyrus. (**C**) Changes in the MTCO1 marker in layer 3 neurons of the middle frontal gyrus. (**D**) Changes in the MTCO2 marker in layer 3 neurons of the middle frontal gyrus. (**E**) Changes in the mitochondrial complex V marker in layer 5 neurons of the middle frontal gyrus. (**F**) Changes in the IF1 marker in layer 5 neurons of the middle frontal gyrus. (**G**) Changes in the MTCO1 marker in layer 5 neurons of the middle frontal gyrus. (**H**) Changes in the MTCO2 marker in layer 5 neurons of the middle frontal gyrus. Asterisks indicate statistically significant differences. Y axis—level of mean intensity ± SD.

In AD, layer 3 of the frontal cortex shows a tendency toward decreased levels of complex V marker compared to the aging group, alongside increased levels of the IF-1 marker (*p* = 0.0043); MTCO1 marker levels decreased relative to the aging group (*p* = 0.0227), while MTCO2 marker levels increased (*p* = 0.0329). In layer 5, despite largely unchanged complex V marker levels, a pronounced increase in the IF-1 marker was observed (*p* = 0.0016); the MTCO1 marker levels decreased relative to the aging group (*p* = 0.0293) (Figure 1), whereas the MTCO2 marker levels showed no significant change (Figure 2A–H).

### 2.2. Anterior Cingulate Cortex

**Figure 3:**
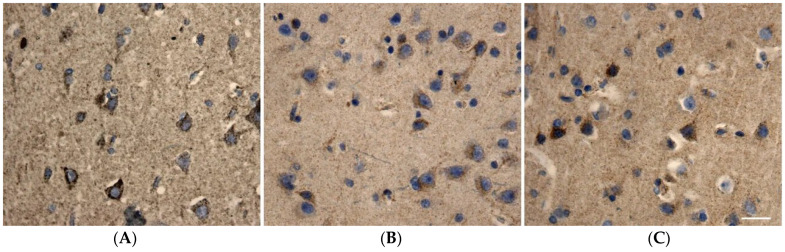
(**A**–**C**) Immunohistochemical distribution of the MTCO2 subunit marker in layer 3 neurons of the anterior cingulate gyrus. (**A**) Staining in young control group neurons; (**B**) staining in aging group neurons; (**C**) staining in AD patient neurons. Objective magnification: 40×. Scale bar = 25 μm.

During aging, neurons of the anterior cingulate gyrus exhibit decreased levels of mitochondrial complex V in both layer 3 (*p* = 0.0031) and layer 5 (*p* = 0.0079) neurons. IF1 factor levels increased in layer 3 (*p* = 0.0314) but showed no difference from young controls in layer 5. MTCO1 marker levels tended to increase in layer 3 and did not differ from young controls in layer 5; MTCO2 marker levels decreased in layer 3 (*p* = 0.0053) and showed no difference from controls in layer 5 (Figure 3).

**Figure 4:**
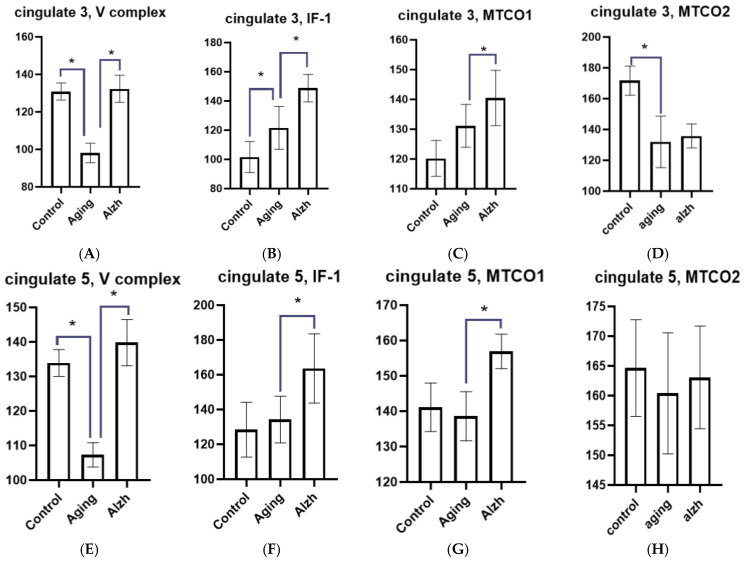
Morphometric analysis results of immunohistochemical distribution of mitochondrial complex V markers, IF1 factor, and mitochondrial complex IV subunits (MTCO1 and MTCO2) in layer 3 and 5 neurons of the cingulate gyrus. (**A**) Changes in the mitochondrial complex V marker in layer 3 neurons. (**B**) Changes in the IF1 marker in layer 3 neurons. (**C**) Changes in the MTCO1 marker in layer 3 neurons. (**D**) Changes in the MTCO2 marker in layer 3 neurons. (**E**) Changes in the mitochondrial complex V marker in layer 5 neurons. (**F**) Changes in the IF1 marker in layer 5 neurons. (**G**) Changes in the MTCO1 marker in layer 5 neurons. (**H**) Changes in the MTCO2 marker in layer 5 neurons. Asterisks indicate statistically significant differences. Y axis—level of mean intensity ± SD.

In AD, changes in neurons were less heterogeneous: a marked increase in complex V marker and IF1 levels was observed in both layers 3 (*p* = 0.0026; *p* = 0.0401) and 5 (*p* = 0.0029; *p* = 0.0421). Both layers also showed elevated MTCO1 levels (*p* = 0.0280; *p* = 0.0375) and no significant change in MTCO2 marker levels (Figure 3 and Figure 4A–H).

### 2.3. Head of the Caudate Nucleus (Basal Ganglia)

**Figure 5:**
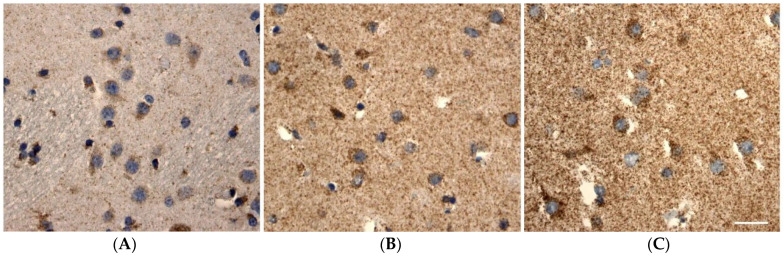
(**A**–**C**) Immunohistochemical distribution of the α-subunit marker of complex V in neurons of the caudate nucleus head. (**A**) Staining in young control group neurons; (**B**) staining in aging group neurons; (**C**) staining in AD patient neurons. Objective magnification: 40. Scale bar = 25 μm.

**Figure 6:**
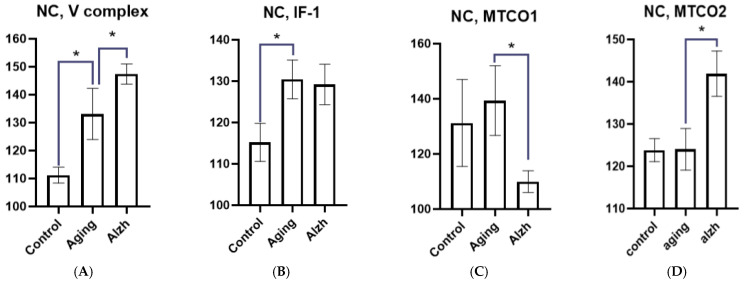
Results of morphometric analysis of immunohistochemical distribution of mitochondrial complex V markers (**A**) factor IF1 (**B**) mitochondrial complex IV subunits—MTCO1 (**C**) and MTCO2 (**D**) in neurons of the caudate nucleus head. Asterisks indicate statistically significant differences. Y axis—level of mean intensity ± SD.

In the head of the caudate nucleus during aging, neurons exhibit an increased amount of complex V (*p* = 0.0057) markers (Figure 5) and factor IF-1 (*p* = 0.0054), while no changes occur in MTCO1 and MTCO2 marker levels. In AD, IF-1 marker levels remain unchanged compared to the elderly group, whereas complex V marker levels increase (*p* = 0.0172) (Figure 5); MTCO1 marker levels significantly decrease (*p* = 0.0326), while MTCO2 marker levels increase (*p* = 0.0041) (Figure 6A–D).

### 2.4. Hippocampus

**Figure 7:**
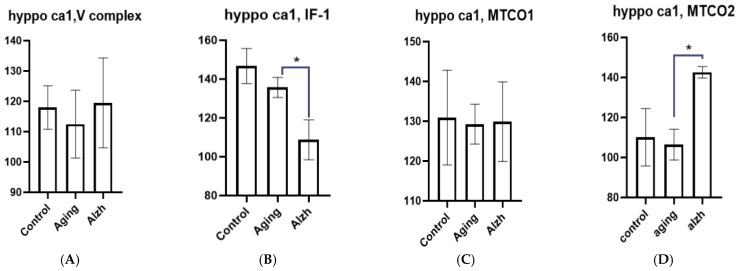
Results of morphometric analysis of immunohistochemical distribution of mitochondrial complex V markers (**A**) IF1 factor (**B**) and complex IV subunits—MTCO1 (**C**) and MTCO2 (**D**) in CA1 hippocampal neurons. Asterisks indicate statistically significant differences. Y axis—level of mean intensity ± SD.

In the CA1 hippocampal neurons during aging, there is a tendency toward decreased IF1 levels against unchanged amounts of complex V markers, MTCO1, and MTCO2 compared to controls. In AD, complex V and MTCO1 marker levels also show no significant change; however, IF1 (*p* = 0.0497) marker levels decrease substantially, while MTCO2 (*p* = 0.0121) increases (Figure 7A–D).

**Figure 8:**
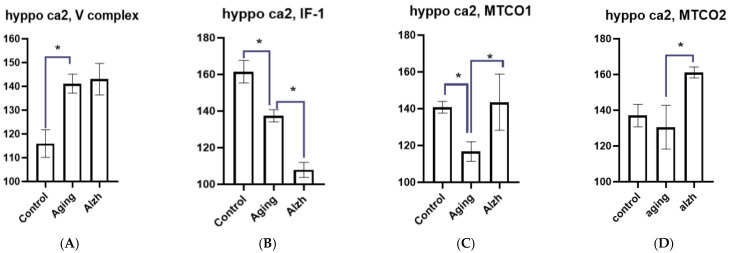
Results of morphometric analysis of immunohistochemical distribution of mitochondrial complex V markers (**A**) IF1 factor (**B**) and complex IV subunits—MTCO1 (**C**) and MTCO2 (**D**) in CA2 hippocampal neurons. Asterisks indicate statistically significant differences. Y axis—level of mean intensity ± SD.

In the CA2 hippocampal neurons during aging, a marked increase in complex V marker (*p* = 0.0114) levels was observed against decreased IF1 (*p* = 0.0145) and MTCO1 (*p* = 0.0484) markers, with MTCO2 levels unchanged from controls. In AD, no changes in complex V marker levels were detected, whereas IF1 marker (*p* = 0.0082) levels decreased significantly, and MTCO1 (*p* = 0.0340) and MTCO2 (*p* = 0.0121) marker levels increased (Figure 8A–D).

### 2.5. Inferior Parietal Lobule

**Figure 9:**
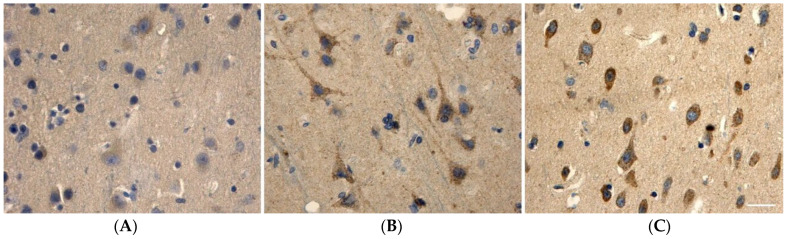
(**A**–**C**) Immunohistochemical distribution of complex V inhibitor marker (IF1) in layer 5 neurons of the inferior parietal lobule. (**A**) Staining in young control group neurons; (**B**) staining in aging group neurons; (**C**) staining in AD patient neurons. Objective magnification: 40×. Scale bar = 25 μm.

During aging, neurons of the inferior parietal lobule exhibit a marked decrease in complex V (*p* < 0.05 all layers) marker alongside an increase in IF1 marker (*p* < 0.05) in layer 2, 3, 4, and 5 neurons (Figure 9). In AD, neurons of layers 2, 3, 4, and 5 in the inferior parietal lobule show increased complex V marker (*p* < 0.05) levels and a significant rise in the IF1 (*p* < 0.05 all layers) marker (Figure 9).

During aging, MTCO1 levels showed no significant change in the neurons of layers 2, 4, and 5 of the inferior parietal lobule but decreased (*p* = 0.0482) in layer 3. MTCO2 marker levels remained unchanged in layer 2 neurons but significantly decreased in layers 3, 4, and 5 neurons (*p* < 0.05) of the inferior parietal lobule.

**Figure 10:**
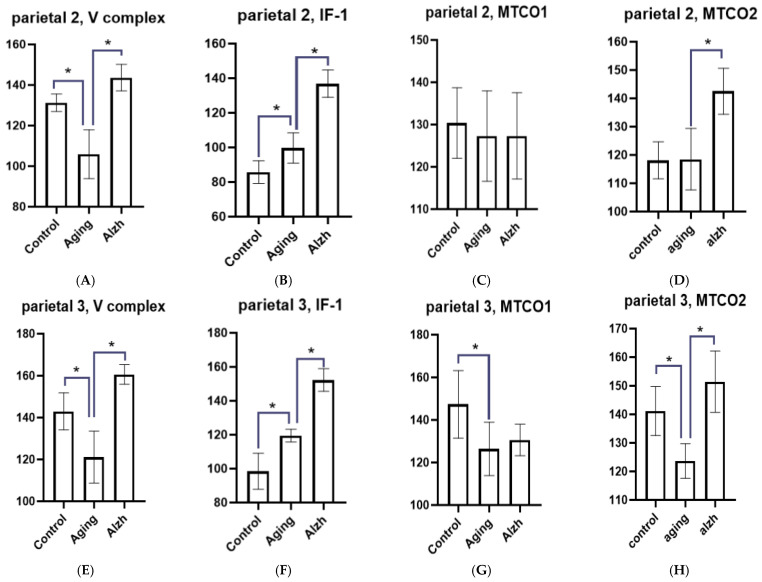

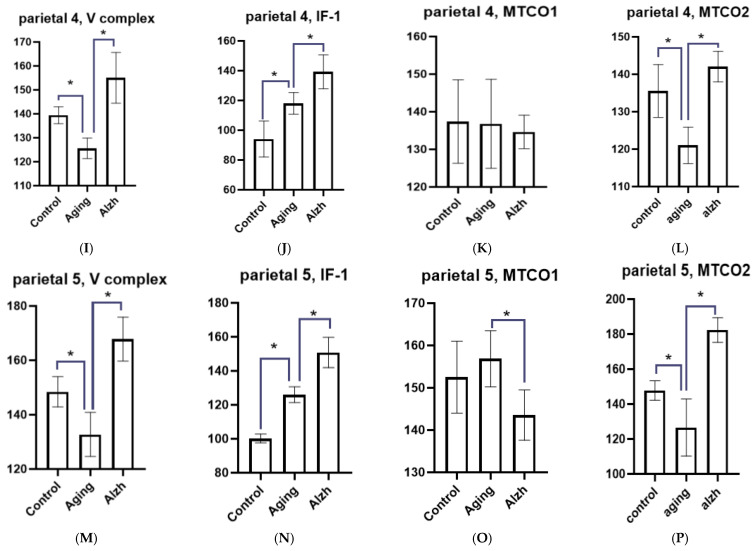
(**A**–**C**) Morphometric analysis results of immunohistochemical distribution for complex V markers (**A**) IF1 factor (**B**) mitochondrial complex IV subunits MTCO1 (**C**) and MTCO2 (**D**) in layer 2 neurons of the inferior parietal lobule. (**E**–**H**) Morphometric analysis results for complex V markers (**E**) IF1 factor (**F**), MTCO1 (**G**), and MTCO2 (**H**) in layer 3 neurons. (**I**–**L**) Morphometric analysis results for complex V markers (**I**) IF1 factor (**J**) MTCO1 (**K**) and MTCO2 (**L**) in layer 4 neurons. (**M**–**P**) Morphometric analysis results for complex V markers (**M**) IF1 factor (**N**) MTCO1 (**O**) and MTCO2 (**P**) in layer 5 neurons. Asterisks indicate statistically significant differences. Y axis—level of mean intensity ± SD.

In AD, MTCO1 levels were unchanged in layer 2, 3, and 4 neurons but decreased in layer 5 (*p* = 0.0462) neurons. MTCO2 levels (*p* < 0.05) increased across all examined layers (Figure 10A–P).

## 3. Discussion

It should be emphasized that immunohistochemistry provides only an indirect assessment of the mitochondrial electron transport chain. While terms such as ‘functional destabilization’ or alterations in ‘energy production’ are invoked as potential explanations for the quantitative changes observed in the proteins studied, these functional parameters were not directly measured.

### 3.1. Mitochondrial Complex IV

Immunohistochemical analysis results for MTCO1 and MTCO2 subunits are significant, as the latter represent two distinct catalytic centers responsible for sequential stages of cytochrome c oxidase function. The MTCO2 subunit, containing a copper-containing dimeric center, accepts and transfers electrons from cytochrome c. The MTCO1 subunit, containing hemes, transfers electrons to oxygen. Thus, their functions are strictly specialized and non-interchangeable. Their combined study allows for not merely quantifying the complex but assessing its functional integrity: an imbalance in these subunits’ levels indicates disrupted cytochrome c oxidase stoichiometry and assembly, which may cause ETC defects and increased reactive oxygen species production.

### 3.2. MTCO1 Subunit

In the neurons of most studied regions (layer 5 of the anterior cingulate gyrus, head of the caudate nucleus, hippocampal CA1 region, layers 2, 4, and 5 of the inferior parietal lobule) during aging, no changes in MTCO1 marker levels were observed, indicating relative stability of this subunit of mitochondrial complex IV. Decreased MTCO1 marker levels were noted in the neurons of the hippocampal CA2 region and layer 3 of the inferior parietal lobule, which may reflect vulnerability of neurons in these regions during aging. This aligns with the literature reports of reduced mitochondrial complex IV subunit levels during aging [26]. Notably, increased MTCO1 marker levels were detected in neurons of layers 3 and 5 of the middle frontal gyrus and layer 3 of the anterior cingulate gyrus during aging, potentially reflecting a compensatory response (Table 1).

In AD, relatively stable MTCO1 marker levels were maintained only in the neurons of the hippocampal CA1 region and layers 2, 3, and 4 of the inferior parietal lobule. Increased MTCO1 marker levels were found in both studied layers of the anterior cingulate gyrus and in hippocampal CA2 region neurons, suggesting possible compensatory changes. Decreased MTCO1 marker levels were observed in both studied layers of the middle frontal gyrus, head of the caudate nucleus, and layer 5 of the inferior parietal lobule, potentially indicating both complex IV functional deficits and impaired electron transport chain activity, exacerbating neuronal oxidative stress (Table 2).

### 3.3. MTCO2 Subunit

During aging, no changes in the levels of the MTCO2 marker were detected in neurons of the majority of examined regions (layers 3 and 5 of the middle frontal gyrus, layer 5 of the anterior cingulate gyrus, head of the caudate nucleus, CA1 and CA2 zones of the hippocampus, layer 2 of the inferior parietal lobule), indicating relative stability of this subunit of mitochondrial complex IV. Decreased levels of the MTCO2 marker were observed in layer 5 of the anterior cingulate gyrus and in layers 3, 4, and 5 of the inferior parietal lobule; this coincided with a reduction in mitochondrial complex V in these regions. The results demonstrating a decrease in the MTCO2 marker in certain brain regions also confirm data on reduced levels of mitochondrial complex IV subunits during aging [26]. Notably, during aging, the MTCO2 marker level did not increase in any examined regions, unlike the MTCO1 marker.

In AD, a relatively stable level of the MTCO2 marker is maintained only in the neurons of layer 5 of the middle frontal gyrus and layers 3 and 5 of the anterior cingulate gyrus. In neurons of all other regions, a significant increase in the levels of the MTCO2 marker is observed, which may represent a compensatory mechanism, particularly against reduced MTCO1 marker levels in several areas (layer 3 of the middle frontal gyrus, head of the caudate nucleus, layer 5 of the inferior parietal lobule). In AD, no decrease in the MTCO2 marker level was detected in any examined regions, unlike the MTCO1 marker.

Thus, both subunits of mitochondrial complex IV maintain relative quantitative stability in most examined brain regions during normal physiological aging. In AD, significant lability and variability in the quantity of MTCO1 and MTCO2 markers are observed, with frequent opposing directional changes that disrupt the required quantitative ratio of complex IV subunits [12,13], likely representing a hypothetical key manifestation of ETC destabilization in AD.

### 3.4. Mitochondrial Complex V

During neuronal aging, a decrease in mitochondrial complex V marker levels has been detected in most studied regions (layer 3 of the middle frontal gyrus, layers 3 and 5 of the anterior cingulate gyrus, layers 2, 3, 4, and 5 of the inferior parietal lobule), suggesting reduced ATP synthesis in neurons of these regions. No changes in mitochondrial complex V marker levels were observed in neurons of layer 5 of the middle frontal gyrus or in the CA1 region of the hippocampus. An increase in mitochondrial complex V marker levels was found in the neurons of the head of the caudate nucleus and the CA2 region of the hippocampus, consistent with the literature data [14] and potentially reflecting compensatory changes in these regions. For instance, enhanced ATP synthase activity has been demonstrated in the hippocampus of aging mice [27]. Additionally, analysis of the human transcriptome during aging has confirmed increased expression of genes encoding ATP synthase subunits in several brain regions, including the hippocampus, putamen, caudate nucleus, frontal cortex, and anterior cingulate cortex [27].

In AD, neurons in layer 3 of the middle frontal gyrus show a tendency toward reduced levels of mitochondrial complex V marker compared to the physiological aging group. No changes in mitochondrial complex V marker levels were detected in layer 5 neurons of the middle frontal gyrus or in the CA1 and CA2 regions of the hippocampus. However, all other examined regions (layers 3 and 5 of the anterior cingulate gyrus, layers 2, 3, 4, and 5 of the inferior parietal lobule, and the head of the caudate nucleus) demonstrated a significant increase in mitochondrial complex V marker levels, which fundamentally distinguishes AD patients from the physiological aging group. This finding is consistent with the literature data: for instance, A.J.T. Yang’s study [28] demonstrated increased ATP synthase levels in the prefrontal cortex of AD patients against reduced oxygen consumption by mitochondrial complex IV, suggesting a compensatory increase in mitochondrial complex expression to maintain mitochondrial function amid metabolic disturbances. Furthermore, studies on the transgenic mouse model line (J20 Tg) expressing a mutant form of amyloid-beta precursor protein corresponding to the Swedish familial form of Alzheimer’s disease demonstrated a 12.2-fold increase in α-subunit expression of ATP synthase in whole-brain homogenates compared to non-transgenic mouse brain homogenates, which the authors attribute to cellular stress responses maintaining energy production [29]. Consequently, the absence of increased mitochondrial complex V quantity in hippocampal neurons in Alzheimer’s disease may indicate more pronounced damage in this region. The increased levels of two mitochondrial complex IV subunits (MTCO1 and MTCO2) in the hippocampal CA2 zone neurons might serve as a compensatory response to decreased ATP synthase levels in these cells. On the other hand, mitochondrial protein accumulation in the hippocampus may be associated with impaired clearance of defective organelles (mitophagy) [29]. Additionally, the study by Cottrell et al. [30] investigated the correlation between mitochondrial complex IV deficiency and AD progression, revealing that hippocampal neurons containing neurofibrillary tangles exhibit high levels of mitochondrial complex IV. The reasons for this remain unclear, and the relationship between neurofibrillary tangles and mitochondria requires further investigation.

### 3.5. The Inhibitor of Mitochondrial Complex V, IF-1

During neuronal aging, an increase in the IF-1 marker was observed in most studied regions (layer 3 of the anterior cingulate gyrus, head of the caudate nucleus, layers 2, 3, 4, and 5 of the inferior parietal lobule), which may indicate pronounced neuroprotective activity of this protein in these regions. No changes were registered in neurons of layer 3 of the middle frontal gyrus and layer 5 of the anterior cingulate gyrus. A decrease in the IF-1 marker was found in the neurons of layer 5 of the middle frontal gyrus and CA1/CA2 hippocampal zones, which may be considered a factor of vulnerability for neurons in these regions.

In AD, neurons exhibit an even more pronounced increase (compared to the aging group) in the IF-1 marker, noted in all studied areas except the hippocampus and head of the caudate nucleus. Importantly, in the head of the caudate nucleus, the IF-1 marker level was elevated relative to the young control group but did not differ from the aging group. The only brain region where a decrease in the IF-1 marker was detected in AD was the CA1 and CA2 hippocampal zones, indicating a pronounced reduction in the neuroprotective effect of IF-1 in AD compared not only to young patients but also to relatively elderly patients. Given the crucial role of IF-1 in regulating cognitive abilities, particularly memory [21], it can be concluded that decreased IF-1 levels in the CA1 and CA2 hippocampal zones may be one of the key links in AD pathogenesis. Furthermore, the demonstrated increase in Drp-1 marker in neurons in AD reported in several publications [31] may promote Bax-dependent apoptosis activation [32,33]. Combined with deficiency of the neuroprotector IF-1 and complex V, along with elevated levels of complex IV as a source of additional free radicals, this may hypothetically lead to neuronal energy collapse and subsequent triggering of neuronal apoptosis in the hippocampus.

Not only are the differential changes in the described ETC markers during aging and AD important, but so is their combined assessment in a specific brain region. Thus, with aging, a decrease in the V complex is detected in both layers of the anterior cingulate gyrus, which could be explained, for example, by decreased mitochondrial mass during aging. However, the amount of MTCO1 in both layers increases, while the amount of MTCO2 does not change in layer 3 neurons and decreases in layer 5 neurons, indicating heterogeneity in the response of ETC proteins. In the middle frontal gyrus, the amount of MTCO1 increased in both layers, while the amount of MTCO2 remained unchanged, and the amount of complex V decreased only in layer 3 neurons. Results in the hippocampus were also heterogeneous: in the neurons of the CA2 region, the amount of complex V increased, MTCO1 decreased, and MTCO2 remained unchanged. No significant changes were observed in the CA1 region. In the neurons of head of the caudate nucleus, the amount of complex V increased, while the amount of MTCO1 and MTCO2 remained unchanged. A decrease in complex V was observed in neurons of all layers of the inferior parietal lobule; however, MTCO1 decreased only in layer 3, while MTCO2 decreased in layers 3, 4, and 5.

Changes in Alzheimer’s disease were also characterized by heterogeneity within each zone. Thus, in neurons of the middle frontal gyrus, a decrease in the V complex in layer 3 and an amount of the V complex in layer 5 did not differ from the control; MTCO1 decreased in both layers, while MTCO2, on the contrary, increased in layer 3 and did not change in layer 5. In the neurons of anterior cingulate gyrus, changes in the V complex and MTCO1 were unidirectional—they increased, but the amount of MTCO2 did not change. In the head of the caudate nucleus, an increase in the V complex was accompanied by an increase in MTCO2, but also by a deficit of MTCO1. Interestingly, a pronounced change in the amount of the V complex in the hippocampus did not occur, whereas the amount of MTCO1 (in CA1) together with MTCO2 (in CA2) increased. In neurons of the inferior parietal lobule, a combined increase in the amount of complex V and MTCO2 was detected, while MTCO1 decreased in layer 5 and did not change in the remaining layers.

Thus, a combined analysis of markers across anatomical zones indicates the absence of a unified compensatory response of ETC proteins in both aging and AD. Opposite changes in two or all three parameters in the described structures may hypothetically indicate an absence of coordinated regulatory adaptation of neurons during aging and neurodegeneration. Interpretation of these results highlights the need to identify factors that selectively affect different respiratory chain complexes (e.g., complex I or IV), as well as the causes that impede the coordinated response of structural proteins even within a single complex.

## 4. Materials and Methods

### 4.1. Study Setting and Tissue Samples

This investigation utilized postmortem brain tissue obtained from the Russian Center of Neurology and Neurosciences (Moscow, Russia). The primary study cohort comprised samples from 18 patients (aged 85–89 years) with confirmed Alzheimer’s disease (Braak stages 3–4), whose cause of death was pulmonary thromboembolism in the presence of post-infarction or atherosclerotic cardiosclerosis associated with hypertension.

For comparative purposes, tissue samples from two control groups were analyzed:

1. Aged Control Group: Individuals over 85 years of age (range: 85–99 years; *n* = 18) without neuropathological signs of AD, who died from analogous cardiovascular causes.
2. Young Control Group: Donors aged 35–45 years (*n* = 18) with a diagnosis of sudden cardiac death.

### 4.2. Exclusion Criteria

Uniform exclusion criteria were applied to all donors across the three groups. These included: acute and prior stroke, neuromuscular diseases, decompensated (severe insufficiency) cardiovascular or other systemic diseases, acute infectious, infectious-allergic, and autoimmune diseases, malignant tumors of any localization at stages III–IV, alcohol and drug misuse. Additionally, the study excluded deceased individuals with asymptomatic hemodynamically significant atherosclerotic stenosis of cerebral arteries (≥70% lumen stenosis), as well as those with a history of grade 3 arterial hypertension.

### 4.3. Tissue Processing and Histology

From pre-fixed brain specimens, standardized tissue blocks (approx. 2–2.5 cm length × 1–1.5 cm width × 0.5–0.7 cm thickness) were dissected from the following anatomical regions: the anterior third of the middle frontal gyrus, the anterior cingulate gyrus, the mid-portion of the caudate nucleus head, the hippocampus (mid-temporal lobe level), and the superior aspect of the inferior parietal lobule. Blocks underwent fixation in 10% neutral buffered formalin for 24 h, followed by routine histological processing through graded alcohols, xylene clearance, and paraffin embedding.

### 4.4. Immunohistochemistry and Microscopy

Serial 3-μm sections were cut on a microtome, placed on positively charged slides (SuperFrost Plus, Thermo Scientific, Waltham, MA, USA), and dried overnight at 48 °C. For general morphological assessment, sections were stained with hematoxylin and eosin (H&E) and by the Nissl method. Immunohistochemical analysis was performed using a panel of four primary antibodies:

1. ATP5H Monoclonal Antibody (7F9BG1); monoclonal; ThermoFisher (Waltham, MA, USA); 459000; dilution 1:200;
2. MTCO2 Polyclonal antibody; polyclonal; Proteintech (Rosemont, IL, USA); 55070-1-AP; dilution 1:200;
3. MTCO1 Monoclonal Antibody (1D6E1A8); monoclonal; ThermoFisher (Waltham, MA, USA); 459600; dilution 1:200;
4. ATPIF1 Monoclonal Antibody (5E2D7); monoclonal; ThermoFisher (Waltham, MA, USA); A-21355; dilution 1:200.

Antibody detection was achieved using the Novolink Polymer Detection System (Leica Biosystems, Nussloch, Germany), a polymer-based peroxidase visualization kit. Serial sections were cut from paraffin blocks. Each antibody was applied to a separate section using chromogenic immunohistochemistry. All four serial sections were then compared.

### 4.5. Image Acquisition and Quantitative Analysis

Digital micrographs were captured using a Leica DMLB microscope (Leica Microsystems GmbH, Wetzlar, Germany) equipped with a Leica DC-300 camera (Leica Microsystems GmbH, Wetzlar, Germany). Quantitative analysis of immunohistochemical staining intensity was conducted on 150 neuronal perikarya per case using Leica Qwin 2.7 software. Regions analyzed included: layers 3 and 5 of the frontal cortex, layers 3 and 5 of the cingulate cortex, basal nuclei region (head of the caudate nucleus), hippocampal CA1 and CA2 areas, and layers 2, 3, 4, and 5 of the parietal cortex. Using a Wacom graphic tablet, the outlines of neuronal bodies and nuclei were traced (excluding nuclear areas). The mean intensity of the outlined cytoplasmic areas of neurons was measured on 8-bit images (0 = white, 255 = black), with subsequent subtraction of background staining values.

### 4.6. Statistical Analysis

Statistical analysis and data visualization were performed using Statistica 13.0 and GraphPad Prism 8.0 software. The normality of data distribution was evaluated using the Shapiro–Wilk test. Intergroup comparisons were made using one-way ANOVA with Fisher’s LSD post hoc test. A *p*-value of less than 0.05 was defined as the threshold for statistical significance.

## 5. Conclusions

Thus, the alterations in mitochondrial chain proteins (subunits of IV and V mitochondrial complexes) and the regulator of the electron transport chain (ETC) IF-1 factor described in this study may indicate profound disorganization of oxidative phosphorylation in neurons in AD, a pattern that fundamentally differs from changes observed in normal physiological aging [9]. Specifically, AD reveals selective regional loss of adaptive mechanisms, most prominently in the hippocampus, characterized by the absence of compensatory increases in ATP synthase quantity and a marked reduction in the levels of the neuroprotective IF-1 factor [9]. Together with elevated subunits of complex IV (a potential source of ROS), this may compromise neuronal energy homeostasis and hypothetically fail to block proapoptotic signals. Conversely, most examined cortical regions exhibit coordinated compensatory responses (increased IF-1 factor alongside elevated complex V markers). However, combined with divergent changes in complex IV subunit levels (which may indicate disrupted stoichiometry and functional activity of this complex), this may represent an attempt to sustain ATP production amid inefficient ETC function. This study establishes that impaired ETC functionality constitutes a crucial pathogenetic mechanism underlying neuronal damage in AD.

## Figures and Tables

**Table 1:** Quantitative changes in studied biomarkers during aging relative to the young group.

Region	Complex V	Complex V Inhibitor (IF-1)	MTCO1	MTCO2
Middle frontal gyrus, layer 3	↓	N	↑	N
Middle frontal gyrus, layer 5	N	↓	↑	N
Anterior cingulate gyrus, layer 3	↓	↑	↑	↓
Anterior cingulate gyrus, layer 5	↓	N	N	N
Head of the caudate nucleus	↑	↑	N	N
Hippocampus, CA1	N	↓	N	N
Hippocampus, CA2	↑	↓	↓	N
Inferior parietal lobule, layer 2	↓	↑	N	N
Inferior parietal lobule, layer 3	↓	↑	↓	↓
Inferior parietal lobule, layer 4	↓	↑	N	↓
Inferior parietal lobule, layer 5	↓	↑	N	↓

The table shows a visual representation of the studied proteins’ changes by using graphical symbols: up arrow—increase compared to the young control group; down arrow—decrease compared to the young control group. N—no significant changes compared to the young control group.

**Table 2:** Quantitative changes in studied biomarkers in AD relative to the aging group.

Region	Complex V	Complex V Inhibitor (IF-1)	MTCO1	MTCO2
Middle frontal gyrus, layer 3	↓	↑	↓	↑
Middle frontal gyrus, layer 5	N	↑	↓	N
Anterior cingulate gyrus, layer 3	↑	↑	↑	N
Anterior cingulate gyrus, layer 5	↑	↑	↑	N
Head of the caudate nucleus	↑	N	↓	↑
Hippocampus, CA1	N	↓	N	↑
Hippocampus, CA2	N	↓	↑	↑
Inferior parietal lobule (layers 2, 3, 4)	↑	↑	N	↑
Inferior parietal lobule, layer 5	↑	↑	↓	↑

The table shows a visual representation of the studied proteins’ changes by using graphical symbols: up arrow—increase compared to the aging group; down arrow—decrease compared to the aging group. N—no significant changes compared to the aging group.

## Data Availability

All the data are shown in the main manuscript.
